# Automated deep learning model for estimating intraoperative blood loss using gauze images

**DOI:** 10.1038/s41598-024-52524-3

**Published:** 2024-01-31

**Authors:** Dan Yoon, Mira Yoo, Byeong Soo Kim, Young Gyun Kim, Jong Hyeon Lee, Eunju Lee, Guan Hong Min, Du-Yeong Hwang, Changhoon Baek, Minwoo Cho, Yun-Suhk Suh, Sungwan Kim

**Affiliations:** 1 Interdisciplinary Program in Bioengineering, Graduate School, Seoul National University, Seoul, 08826 Korea; 2 Department of Surgery, Seoul National University Bundang Hospital, Seongnam, 13620 Korea; 3 Department of Surgery, Chung-Ang University Gwangmyeong Hospital, Gwangmyeong, 14353 Korea; 4 Department of Transdisciplinary Medicine, Seoul National University Hospital, Seoul, 03080 Korea; 5 Department of Surgery, Seoul National University College of Medicine, Seoul, 03080 Korea; 6 Department of Biomedical Engineering, Seoul National University College of Medicine, Seoul, 03080 Korea; 7 Institute of Bioengineering, Seoul National University, Seoul, 08826 Korea; 8 Artificial Intelligence Institute, Seoul National University, Seoul, 08826 Korea

**Keywords:** Biomedical engineering, Medical imaging, Gastrointestinal bleeding, Gastric cancer

## Abstract

The intraoperative estimated blood loss (EBL), an essential parameter for perioperative management, has been evaluated by manually weighing blood in gauze and suction bottles, a process both time-consuming and labor-intensive. As the novel EBL prediction platform, we developed an automated deep learning EBL prediction model, utilizing the patch-wise crumpled state (P-W CS) of gauze images with texture analysis. The proposed algorithm was developed using animal data obtained from a porcine experiment and validated on human intraoperative data prospectively collected from 102 laparoscopic gastric cancer surgeries. The EBL prediction model involves gauze area detection and subsequent EBL regression based on the detected areas, with each stage optimized through comparative model performance evaluations. The selected gauze detection model demonstrated a sensitivity of 96.5% and a specificity of 98.0%. Based on this detection model, the performance of EBL regression stage models was compared. Comparative evaluations revealed that our P-W CS-based model outperforms others, including one reliant on convolutional neural networks and another analyzing the gauze’s overall crumpled state. The P-W CS-based model achieved a mean absolute error (MAE) of 0.25 g and a mean absolute percentage error (MAPE) of 7.26% in EBL regression. Additionally, per-patient assessment yielded an MAE of 0.58 g, indicating errors < 1 g/patient. In conclusion, our algorithm provides an objective standard and streamlined approach for EBL estimation during surgery without the need for perioperative approximation and additional tasks by humans. The robust performance of the model across varied surgical conditions emphasizes its clinical potential for real-world application.

## Introduction

Estimated blood loss (EBL) serves as a pivotal parameter in surgery, offering crucial insights into fluid management, blood transfusion decisions, and ultimately influencing postoperative outcomes and complications^1,2^. The meticulous monitoring of EBL is considered as a quality marker in perioperative care^3–6^. However, the reliability of existing methods for estimating intraoperative blood loss is compromised by subjectivity, relying heavily on the visual assessments made by the operating staffs^7–10^. Such visual estimations are susceptible to personal biases, variations in experience, and individual differences in estimation capabilities^11–14^, often resulting in underestimation of the actual blood loss and, consequently, potential risks and adverse outcomes for patients^9,15,16^.

While EBL can be estimated through changes in hematocrit and vital signs during the operation, this method does not guarantee the measurement of the absolute volume of blood loss^17,18^. The gold standard of accurate EBL quantification involves weighing the surgical gauze used to absorb the blood in the surgical field and measuring the volume in the suction bottle. This method, applicable in both open surgeries and minimally invasive surgeries (MIS), is particularly advantageous in MIS due to reduced trauma to the patient’s body^19^. Laparoscopy, a prevalent form of MIS, inherently has restricted surgical visibility due to the narrow operation field^20^, making conventional blood loss estimation challenging^21^.

The current method for estimating blood loss in laparoscopic surgery entails summing the weight of the used gauzes and the fluid aspirated by the suction device. Unfortunately, this method is inconvenient and labor-intensive, requiring additional manpower, and is not conductive to establishing a real-time intraoperative blood loss monitoring system^22^. Consequently, there is a need for an objective and automated EBL estimation during surgery.

With the advent of artificial intelligence (AI), recent studies have explored automated EBL measurement techniques^23–25^. Some studies classified blood loss into four levels using surgery videos, and others focused on EBL regression by assessing blood areas in images of fully spread gauze using simulated data^23–25^. However, these approaches have limitations, either providing unquantifiable estimates owing to a four-class classification system or lacking real-time capabilities, demanding additional tasks unrelated to the surgical procedure^26,27^. Moreover, these studies did not integrate actual surgical details with blood volume loss, limiting their practical applicability in surgical environments.

This study introduces an innovative approach for automated EBL prediction, utilizing images of gauze obtained from laparoscopic surgical sites. Our method employed a convolutional neural network (CNN), a technique extensively employed for extracting meaningful visual features from images^28–30^ and analyzing the obtained gauze images. The significant contribution of our work is its capacity to furnish quantifiable and reliable estimates of intraoperative blood loss without the need to spread gauzes, allowing analysis in their natural crumpled state. The model is comprehensively trained on an animal experimental dataset and validated on a human patient dataset, ensuring its robustness and clinical applicability. This automated EBL prediction system provides accurate estimates, reducing the reliance on manual measurements and additional intraoperative tasks. Furthermore, the system has the potential to enhance surgical procedures by offering more efficient and patient-safe methods, facilitating precise and dependable monitoring of critical intraoperative factors.

## Materials and methods

### Study design

**Figure 1:**
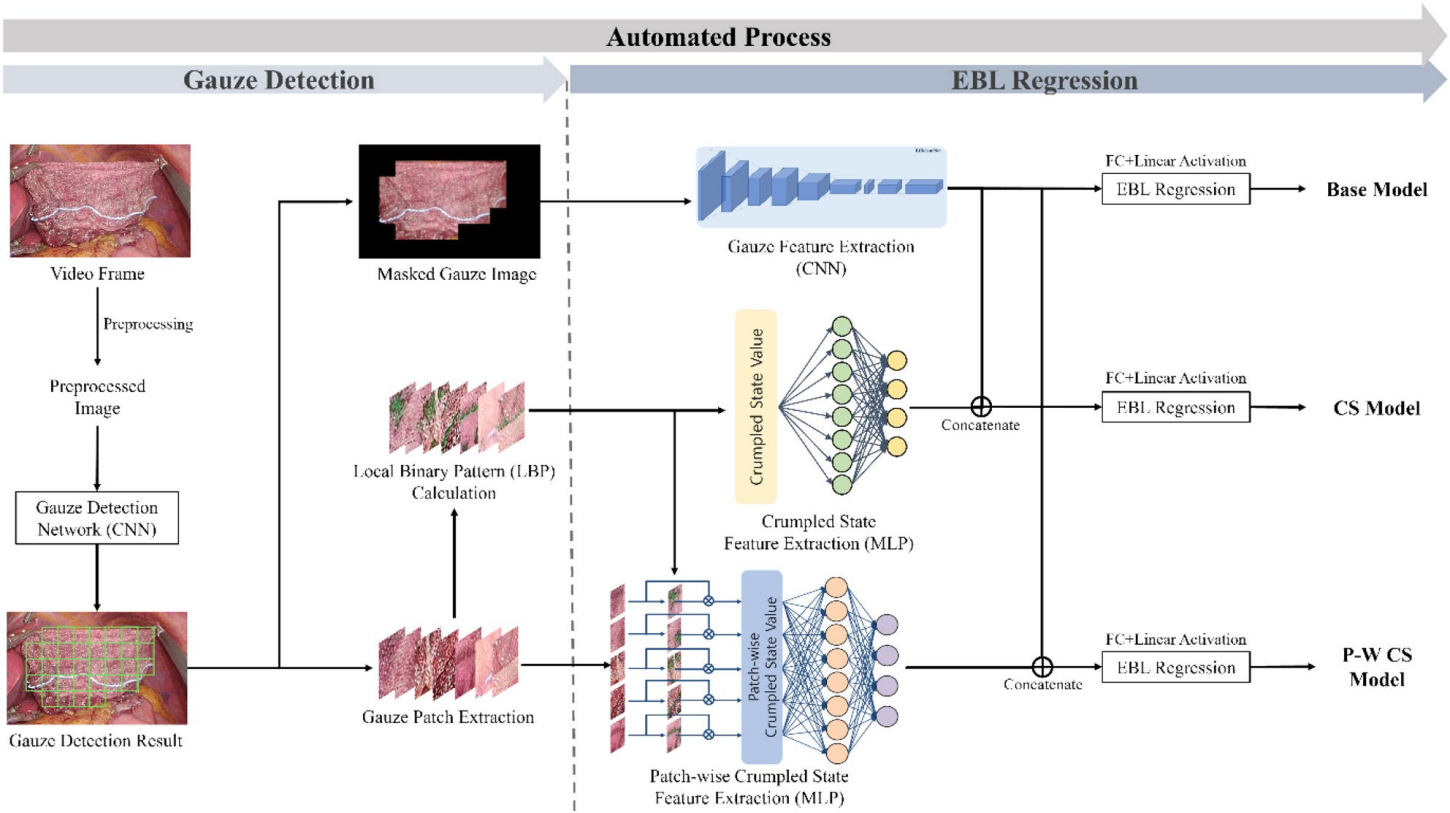
Overall scheme of the gauze EBL prediction includes the gauze detection and EBL regression processes. In the gauze detection stage, the masked gauze image and gauze patches are produced based on the detection result of the input gauze image. Features extracted from these masking images and the crumpled state value (CSV), calculated using local binary pattern (LBP), are concatenated for EBL regression. Abbreviations: CNN, Convolutional neural network; MLP, Multilayer perceptron; FC, Fully connected; CS, Crumpled state; P-W CS, Patch-wise crumpled state.

EBL is defined as the sum of the blood weight absorbed by the gauze. The proposed EBL prediction model includes two stages (Fig. 1). In the gauze detection phase, the process commences with the preprocessing of input images, followed by automated detection of gauze for the extraction of patches and masking in gauze images. This step is crucial for identifying gauze areas within laparoscopic images, setting the stage for further analysis. During the EBL regression stage, feature extraction and the implementation of EBL regression layers are applied. Crumpled state values (CSV) are computed from the identified gauze patches utilizing a texture analysis method, specifically designed for extracting crumpled state features. These extracted features are then concatenated and fed into the EBL regression layers for analysis. The entire process, from video frame extraction to EBL regression, is executed automatically.

### Dataset

To overcome the limited distribution of human data and enhance the robustness of the model, we conducted initial training on a porcine dataset. This dataset was collected in an environment physiologically analogous to that of humans, providing a diverse range of scenarios for training. Subsequently, the model’s performance was assessed using an independent dataset composed of human patients. This approach allowed for the validation of the model’s applicability and accuracy in real-world clinical settings.

#### Porcine dataset

We aimed to develop a consistent model of overall weight distribution using an animal experimental dataset, consisting of 1208 still images from 310 gauze samples. The porcine dataset was obtained through an experiment in which surgeries were performed under laparoscopic conditions identical to human operations (Supplementary Fig. S1). We conducted the experiment on live animals under general anesthesia to collect data for in vivo blood loss measurement. The preclinical experiment using the porcine model was approved by the Institutional Animal Care and Utilization Committee of Seoul National University Bundang Hospital (IACUC No. BA-2303-363-002-01). All experiments were performed in accordance with relevant guidelines and regulations.

Two healthy three-way crossbred female pigs weighing approximately 30 kg underwent laparoscopic surgery at the Preclinical Center of Seoul National University Bundang Hospital on April 21, 2023. Three surgeons specializing in laparoscopic gastrointestinal surgery at Seoul National Bundang University Hospital participated in the study. The surgeons intentionally injured the omental vessels, liver, spleen, or other organs to cause bleeding in the operation field, and then cleared blood using gauze. The gauze was extracted from the body through the trocar after a still shot of the spread gauze was taken in the abdominal cavity, and its weight was calculated using a digital scale. Pigs were monitored by a veterinarian and euthanized according to animal experimental ethics when their vital signs became unstable owing to hypovolemic shock. This study is reported in accordance with ARRIVE guidelines^31^.

#### Patient dataset

The study protocol adhered to the ethical guidelines of the 1975 Declaration of Helsinki and its subsequent revisions and was approved by the institutional review board of Seoul National University Bundang Hospital (IRB No. B-2208-773-302).

Prospective data were collected with the informed consent of patients who underwent laparoscopic gastrectomy for gastric cancer between April 2022 and June 2023 at Seoul National University Bundang Hospital. 473 gauze images were collected from 102 patients who were enrolled in the study. The inclusion criteria were patients over 19 years undergoing laparoscopic gastrectomy. Patients who did not require a gauze during surgery or who underwent open conversion surgery were excluded. Surgical videos were recorded throughout the procedure. Each gauze sample was taken as a still frame and removed through a trocar for weight measurement using a digital scale. Patient clinical data, including demographics, surgical outcomes, and complications, were analyzed.

### Material

All operations were performed using the same model of the laparoscopic device, including the imaging system (IMH-20, Olympus, Tokyo, Japan) and the laparoscope (Endoeye Flex 3D, Olympus, Tokyo, Japan), to equalize surgical settings, such as recording quality, light source, and operation view. The videos were recorded at a resolution of 1920 × 1080 pixels at 60 frames per second.

The surgical gauze was the same product (DaeHan Medical Supply Corporation, Chungju-si, Republic of Korea) of 10 cm × 7.5 cm × 4P, with an average weight of 1.438 g (± 0.01 g).

The digital scale was an Electronic Scale (14,192-641C, IT Caster Ltd., Wan Chai, Hong Kong) with an error range of 0.01 g.

The gauzes were from the abdominal cavity through the laparoscopic 12 mm-sized trocars (Kii^®^ Optical Access System, Applied Medical Resources Corporation, California, USA) or multi-channel glove port (UP04FLV2-B, UNI-PORT, Dalim Corporation, Seoul, Republic of Korea).

### Development of the EBL prediction model

This section discusses the methodology employed in the development of the EBL prediction model. The model encompasses two principal stages: the gauze detection stage and the EBL regression stage, as depicted in Fig. 1. During the gauze detection stage, gauze areas within the surgical field are identified and extracted as both masked gauze images and distinct gauze patches. These extracted images are subsequently processed through the EBL regression stage, which facilitates the prediction of EBL.

#### Training gauze detection model

**Figure 2:**
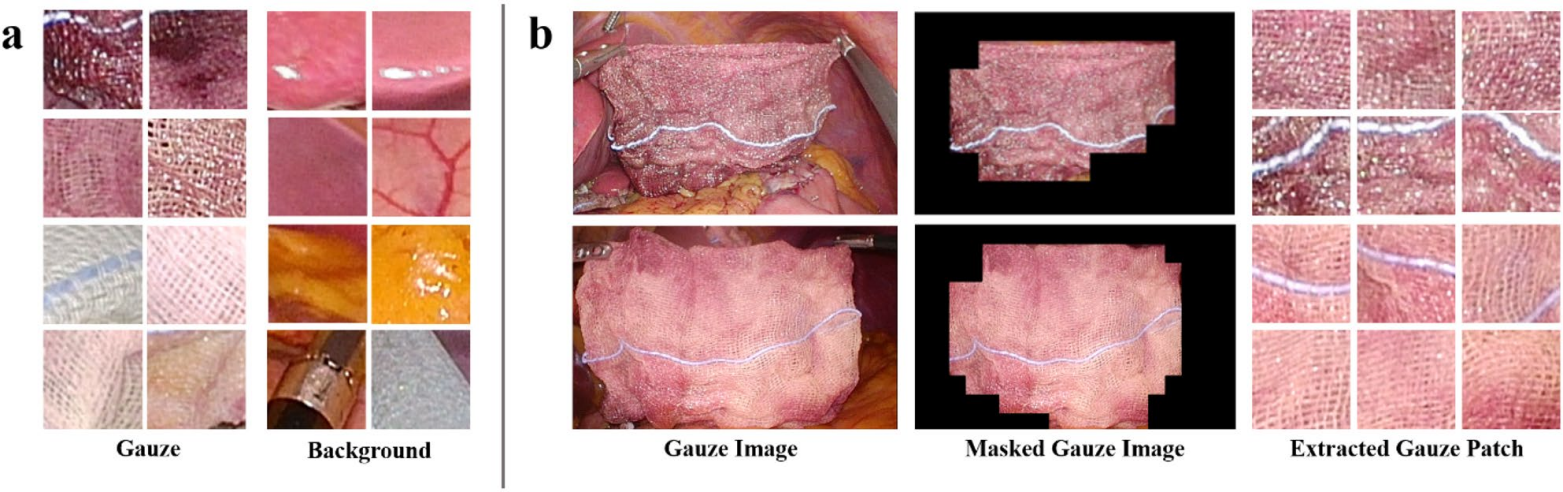
Gauze images used in the development of the EBL regression model. (**a**) Laparoscopic surgery tile images included in the gauze detection dataset, (**b**) preprocessed gauze images including masked images and extracted patches input to the EBL regression network.

We applied the CNN algorithm to develop an automatic gauze-detection system capable of extracting masked gauze images and gauze patches. In this study, we employed EfficientNet, known for achieving efficient results through uniform scaling of depth, width, and resolution^32^. Of the eight models between B0 and B7, we applied EfficientNet B3 and B5 for gauze detection to analyze surgical videos in real-time. Swish was used as the activation function instead of a rectifier linear unit (ReLU) in EfficientNet^33^. To train the gauze detection algorithm, we used the Mendeley dataset^34^, created in a laparoscopic simulator using animal internal organs. It comprises 2935 background tissue blocks, and 1070 gauze tiles presented in three states: clean, stained, and soaked. (Fig. 2a). For each class, the dataset was randomly split in a ratio of 3:1:1 for training, validation, and testing.

Following successful gauze detection, masked gauze images were generated for input into the gauze feature extraction layers, and gauze patches were extracted for calculating the CSV (Fig. 2b). The performance metrics employed were sensitivity, specificity, and precision.

#### Gauze EBL regression network

The prediction of EBL is achieved by processing both masked gauze images and gauze patches through the feature extraction backbone and EBL regression layers within the gauze EBL regression network. Given that gauze used in actual surgical procedures is not typically laid out flat, an analysis of its crumpled state is essential for enhancing EBL measurement accuracy. The model integrates features extracted from the masked gauze images with numerical data derived from the gauze’s crumpled state. These combined inputs are then fed into the regression network to predict EBL. Figure 1 depicts three distinct strategies employed in gauze EBL regression:

(i) Basis regression network based on a masked gauze image (base model).
(ii) Regression network with multiple inputs, including masked gauze images and crumpled state values (CS model).
(iii) Proposed regression network with multiple inputs comprising the patch-wise crumpled state of the gauze and masked gauze image (P-W CS model).

The three models were trained on the porcine dataset to overcome the limited distribution of human data and develop a robust system capable of handling diverse conditions.

The basis regression network consists of a feature extraction backbone, which receives masked gauze image input, and regression layers. Following a series of performance comparison experiments with VGG16 and GoogLeNet, EfficientNetB5 was chosen for the feature extraction backbone. The regression layers included a fully connected (FC) layer and a linear activation function. To reflect the crumpled state of the gauze, we calculated the CSV based on the local binary pattern (LBP) method which is a traditional descriptor for texture analysis^35^. The LBP offers local representation of texture and is determined for every pixel in an image, comparing it with neighboring pixels. A binary value is ascertained for each pixel based on the intensity contrast with its surrounding pixels (radius *r* and number *p*)*.* Specifically, the value 1 is designated when the center pixel’s intensity is less than that of the adjacent pixel, and 0 otherwise. Subsequently, this binary number is converted to its decimal equivalent that is the LBP value for the central pixel as defined in the equation below (1):

1 $$LBP_{P, R} = \mathop \sum \limits_{P = 0}^{P - 1} s\left( {g_{p} - g_{c} } \right)2^{p} ,\quad s\left( x \right) = \left\{ {\begin{array}{*{20}c} {1,\;x \ge 0} \\ {0,\;x < 0} \\ \end{array} } \right.$$

where $g_{c}$ is the intensity value of center pixel and $g_{p}$ denotes the neighboring pixels arranged along a circle with radius *r*^36^*.* The CSV is ascertained by aggregating all the LBP values within the gauze area (with *r* = 3 and *p* = 8), and this aggregate is then normalized by the pixel count in the detected gauze region, as illustrated in Eq. (2).

2 $${\text{Crumpled}}\;{\text{state}}\;{\text{value}}\left( {{\text{CSV}}} \right) = \frac{{{\text{Sum}}\;{\text{of}}\;{\text{gauze}}\;{\text{LBP}}\;{\text{values}}}}{{{\text{Total}}\;{\text{pixels}}\;{\text{of}}\;{\text{detected}}\;{\text{gauze}}\;{\text{region}}}}$$

The calculated CSV was input into a multilayer perceptron (MLP) and concatenated with the output of the gauze feature extraction backbone. The gauze EBL was predicted using the regression layers. However, this CSV did not consider the patch-wise difference of the gauze; therefore, we constructed another EBL regression network based on the P-W CS of the gauze.

To precisely capture the patch-specific blood absorption, the CSV of each gauze patch was weighted according to the EBL associated with that patch. This adjustment was based on the premise that crumpled areas in patches with higher EBL are indicative of greater blood absorption. These calibrated CSVs were then input into the MLP, and their output was concatenated with the results from the gauze feature-extraction backbone. Subsequently, the gauze EBL value was predicted using the regression layer.

### Statistical analysis

Following initial training on the porcine dataset, we validated our algorithm using human patient data to ascertain its real-world applicability. We employed the mean absolute error (MAE) as the regression loss function (3), and the mean absolute percentage error (MAPE) as the performance metric (4)^37^. For performance comparison, we trained using the same dataset and parameters (${\text{batch}}\;{\text{size}} = 32$, ${\text{learning}}\;{\text{rate}} = 5 \times 10^{ - 3}$) by changing the activation function, loss function, and feature-extraction backbone of the basis regression network. VGG16 and GoogLeNet were adapted to the feature extraction network for comparison with EfficientNet-B5 with the MAE and mean squared error (MSE) (5) for the linear and ReLU activation functions^38,39^.

3 $${\text{Mean}}\;{\text{absolute}}\;{\text{error}}\left( {{\text{MAE}}} \right) = \frac{1}{n}\mathop \sum \limits_{i = 1}^{n} \left| {y_{i} - \hat{y}_{i} } \right|$$

4 $${\text{Mean}}\;{\text{absolute}}\;{\text{percentage}}\;{\text{error}}\left( {{\text{MAPE}}} \right) = \frac{1}{n}\mathop \sum \limits_{i = 1}^{n} \left\| {\frac{{y_{i} - \hat{y}_{i} }}{{y_{i} }}} \right\| \times 100$$

5 $${\text{Mean}}\;{\text{squared}}\;{\text{error}}\left( {{\text{MSE}}} \right) = \frac{1}{n}\mathop \sum \limits_{i = 1}^{n} \left( {y_{i} - \hat{y}_{i} } \right)^{2}$$

where $(\hat{y}_{i} ){ }$ and $(y_{i} )$ are the predicted and actual values, respectively, and $n$ is the number of data points.

## Results

To assess the robustness and practical utility of our EBL prediction model, comprehensive evaluations were conducted using both porcine and human datasets. These evaluations, encompassing a range of EBL values, aimed to ascertain the model’s performance under varying EBL conditions. Furthermore, specific per-patient EBL evaluations, crucial in real-world surgical scenarios, were carried out in the clinical study section.

### Preclinical study

**Figure 3:**
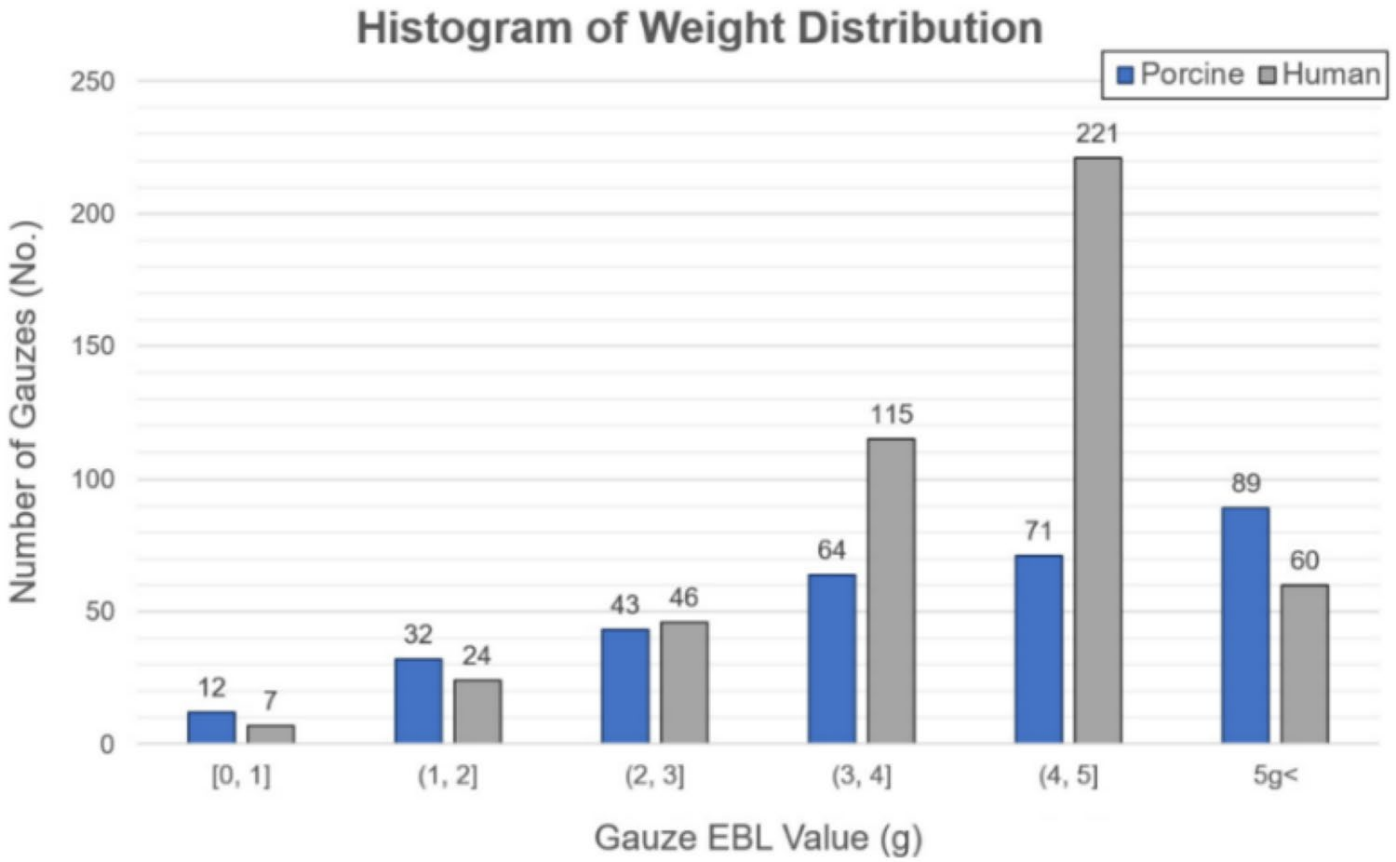
Histogram of weight distribution of the EBL datasets: porcine EBL dataset (blue) and human EBL datasets (gray).

In the preclinical study section, we discuss the outcomes of gauze detection and EBL prediction using the porcine dataset. To validate the model’s performance across diverse EBL ranges, a porcine experiment was conducted in a setting mirroring human surgical procedure, facilitating the collection of relevant datasets. The porcine dataset, consisting of 1208 still images from 310 gauze samples, was similar to the human dataset in terms of color, lighting, and overall appearance (Supplementary Fig. S2). The porcine dataset was randomly divided at a ratio of 3:1:1 to compose the training, validation, and test datasets while considering the weight distribution of the gauze. The weight distributions of the two datasets are shown in Fig. 3. The EBL in the collected porcine data was in the range 0.13–9.98 g, with an average value of 4.17 g.

#### Gauze detection performance of the EBL prediction models

**Figure 4:**
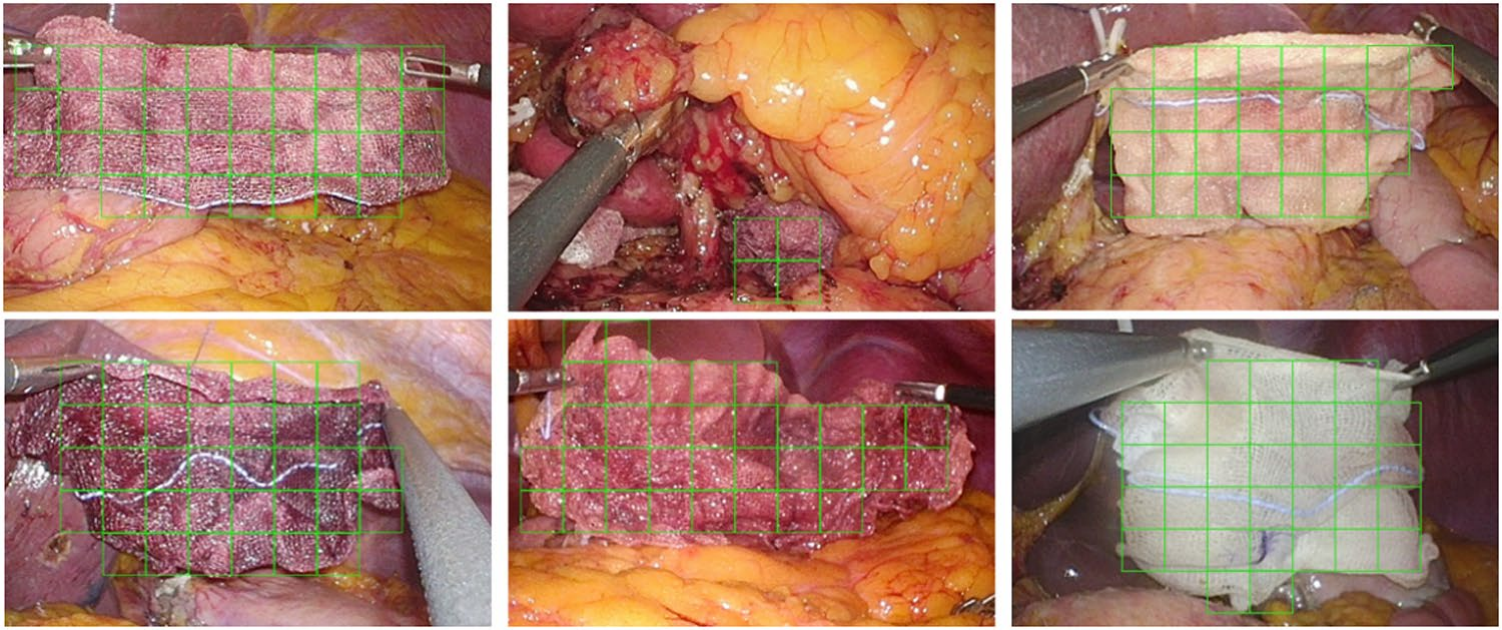
Detection results of EfficientNet-B5 on laparoscopic surgery images from human data.

For effective EBL prediction utilizing gauze images, the extraction of both masked gauze images and gauze patches through the gauze detection process is necessary. The evaluations of the gauze detection model are outlined as follows. We evaluated the gauze detection models with a test set, including 200 gauze patches and 200 background patches from the Mendeley dataset^34^. EfficientNet B3 and B5 exhibited high performance for every metric (Supplementary Table S1). We masked the gauze images and extracted gauze patches using EfficientNet-B5, which showed a higher sensitivity of 96.5%, with a specificity of 98.0% (Fig. 4).

#### EBL prediction performance of the EBL regression networks

The EBL prediction performance was evaluated by inputting the masked gauze image and gauze patches, extracted through the gauze detection model based on EfficientNet-B5, into the regression network for each gauze image. To compare the performances of various regression networks, we trained the basis regression network with three feature-extraction backbones: EfficientNet-B5, VGG16, and GoogLeNet. ReLU and linear activation functions were adapted for each network, and MSE was employed as a loss function for comparison with MAE. All performances were evaluated with fivefold cross-validation. The regression network that employed EfficientNet-B5 as the feature-extraction backbone, linear activation function, and MAE exhibited the highest performance with a MAPE of 8.6% (Table 1). To compare the performance improvement achieved by incorporating the CSV, all networks of the three models—base model, CS model, and P-W CS model—were constructed using EfficientNet-B5 with linear activation and MAE loss function, which exhibited the highest performance.

**Table 1:** Performance of gauze EBL regression networks with three feature extraction backbones.

	Linear activation		ReLU activation	
	MAE	MSE	MAE	MSE
MAPE performance of the gauze EBL regression networks (%)				
EfficientNet-B5	**8.6**	9.5	11.2	13.2
VGG16	10.5	11.1	12.3	13.2
GoogLeNet	13.4	13.2	14.5	16.3

EBL, Estimated blood loss; MAPE, Mean absolute percentage error; MAE, Mean absolute error; MSE, Mean squared error; ReLU, Rectified linear unit.

Significant values are in bold.

To assess the influence of incorporating the gauze-crumpled state into the gauze EBL regression, we performed analyses on the porcine test set, comprising 62 gauzes, using three distinct regression models: the base model, the CS model, and the P-W CS model. These evaluations were conducted per gauze EBL unit to verify performance relative to gauze EBL values. The regression models that incorporated the CSV of the gauze achieved higher performance compared to the base model, showing an improvement of approximately 18.57% across the entire EBL range, as indicated in Table 2. Notably, for gauzes with large EBL values exceeding 5 g, the performance improvement was significant, increasing from 9.10 to 6.15%, an approximate enhancement of 32.42%. However, a slight increase in MAPE was observed in the gauze with an EBL below 3 g in the CS model. This increase is attributed to the minimal influence of the crumpled part in gauzes with lower EBL. Therefore, we developed a network (P-W CS model) that differentially assigns CSV for each EBL value. This modification resulted in a reduced MAPE for all gauze EBL values, as shown in Table 2. In particular, the P-W CS model exhibited a performance improvement of around 25.80% in the lower EBL range (up to 3 g) compared to the CS model, demonstrating a high EBL prediction performance across all EBL ranges. Remarkably, the model, initially developed with porcine data, showed promising results when applied to the human patient dataset, underscoring its robustness and potential for application in real-world clinical settings.

**Table 2:** Performance of the gauze EBL regression models on the porcine test set.

	Base model		CS model		P-W CS model	
	MAE (g)	MAPE (%)	MAE (g)	MAPE (%)	MAE (g)	MAPE (%)
Performance of the gauze EBL regression models evaluated on the test set (n = 62)						
EBL value (x)						
${\text{x}}\le 3{\text{g}}$	0.31	10.36	0.28	9.38	0.21	6.96
$3{\text{g}}<{\text{x}}\le 5{\text{g}}$	0.38	7.06	0.32	5.98	0.23	4.34
$\mathrm{ x}>5{\text{g}}$	0.71	9.10	0.46	6.15	0.43	5.72
Average	0.46	8.56	0.35	6.97	**0.28**	**5.46**

EBL, Estimated blood loss; CS, Crumpled state; P-W CS, Patch-wise crumpled state; MAE, Mean absolute error; MAPE, Mean absolute percentage error.

Significant values are in bold.

### Clinical study

The feasibility of the model’s real-world application was confirmed through the evaluation of the EBL in patients between April 2022 and June 2023; 102 patients were enrolled in the study, and 473 gauze images were collected for the human dataset (Supplementary Table S2). Distal gastrectomy and D1 + lymph node dissection were performed frequently (73.5% and 64.7%, respectively) (Supplementary Table S3). Patients were commonly diagnosed at pathological stage IA stomach cancer (75.5%). No postoperative complications related to bleeding were observed (Supplementary Table S4). The complications classified above Clavien-Dindo grade III were 3 cases among 102 patients (2.9%), including those with duodenal stump leakage, intra-abdominal abscess, and motility disorder.

**Figure 5:**
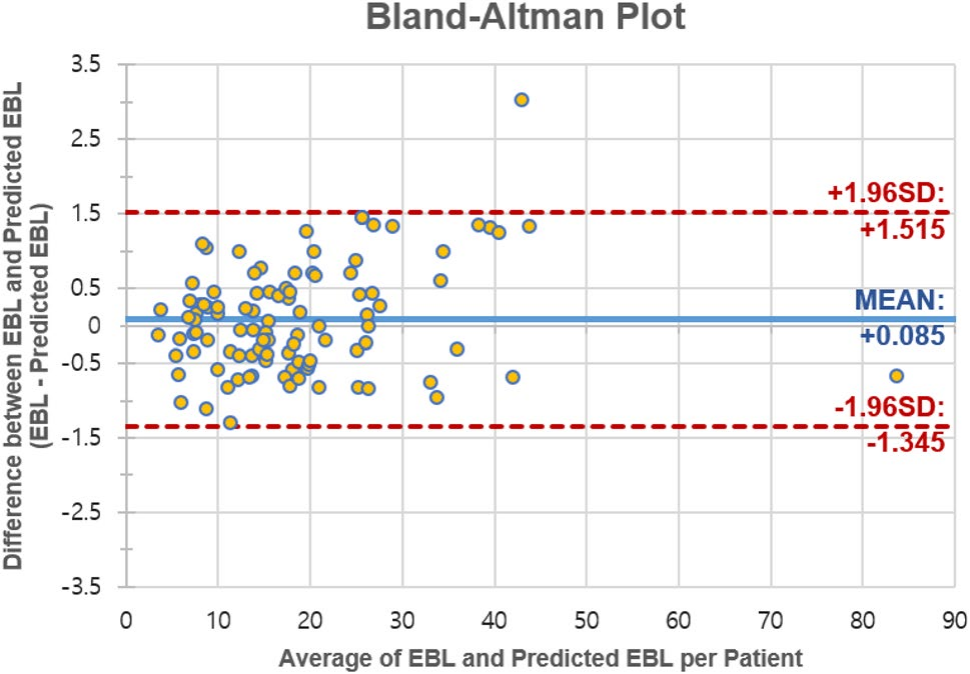
Bland–Altman plot of EBL values per patient. The plot shows the evaluation of EBL for each patient in the human dataset.

To evaluate the efficiency of the P-W CS model using real human data, we implemented both per-gauze and per-patient assessments. In the per-patient evaluation, we aggregated the per-gauze evaluation scores for each individual patient and then compared these totals with the patient’s overall EBL value. The developed P-W CS model demonstrated high performance on human patient data, achieving an MAE of 0.25 g and MAPE of 7.26% (Table 3). In addition, when evaluated on a per-patient basis, the model showed a MAE of 0.58 g and MAPE of 3.64%, indicating errors of less than 1 g. In particular, for large EBL values, such as 83.3 g, the model exhibits a small error of 0.67 g as shown in Fig. 5. Therefore, the model developed based on large-weight porcine data performs well, even for large EBL values of human patients.

**Table 3:** Performance of the gauze EBL regression models on the human patient dataset.

	P-W CS model	
	MAE (g)	MAPE (%)
Performance of the P-W CS model evaluated on the human patient dataset (n = 473)		
EBL value (x)		
$\mathrm{ x}\le 3{\text{g}}$	0.21	15.73
$3{\text{g}}<{\text{x}}\le 5{\text{g}}$	0.22	5.27
${\text{x}}>5{\text{g}}$	0.42	7.67
Average	**0.25**	**7.26**

EBL, Estimated blood loss; CS, Crumpled state; P-W CS, Patch-wise crumpled state; MAE, Mean absolute error; MAPE, Mean absolute percentage error.

Significant values are in bold.

## Discussion

This study aimed to develop an automated EBL prediction system that operates on laparoscopic crumpled gauze images and employs a regression network incorporating P-W CS values. Our system offers more accurate and objective EBL measurements, utilizing solely the gauze images as input data. The application of the P-W CS value significantly enhanced EBL accuracy, especially for folded gauze, thereby obviating the necessity to unfold the gauze completely. Significantly, the model showed high performance across both animal and human datasets, demonstrating robustness and real-world applicability.

Our system constitutes a fully automated workflow that employs crumpled gauze images extracted from surgical videos for the detection of gauze and the subsequent estimation of absorbed blood loss. The gauze detection models accurately identified gauze patches in various surgical conditions (Fig. 4), with a sensitivity of 96.5%, providing reliable inputs for the subsequent EBL regression networks. It can be expected that this system can automatically spot the gauzes and count the number of gauzes during surgery not to be left in the operation field.

Based on the base network, we improved the performance by incorporating the CSV to reflect the degree of gauze crumpling. This enhancement in the CS model was particularly effective in higher EBL, leading to an overall improvement on the test set in MAE of 23.9% and MAPE of 18.6% compared to the base model. To address the potential overestimation of EBL in gauze regions with less absorbed blood due to CSV, we constructed the P-W CS model with differentially weighted CSVs for each patch. This approach yielded the best performance, especially for lower EBL ($\le \hspace{0.17em}$3 g), with a notable enhancement of approximately 25.0% in MAE (Table 2). These results demonstrate the potential for a precise EBL prediction model across the entire EBL weight range.

Our dual-data study, utilizing animal experiments and human patient datasets, exhibited robust results, demonstrating the versatility and reliability of our model across various environments. That is because patient data and porcine data showed similarities in terms of the intra-abdominal environment, blood color, lighting conditions, and other factors influencing the image (Supplementary Fig. S2). Based on a porcine dataset, our developed models achieved robust performance for various EBL values with additional features. Additionally, the P-W CS model exhibited commendable performance on a human validation dataset including 473 gauzes as shown in Table 3, and the per-patient evaluation confirmed the low error under 1 g in the end result used in actual clinical settings demonstrating the practical applicability and the reliability of our approach (Fig. 5).

A notable distinction of our study is the model development using crumpled gauze images sourced from real surgery videos within the abdominal cavity, resulting in high performance. In contrast, previous studies relied on simulated data, artificially saturating gauze with a predetermined volume of blood in an external environment and necessitating gauze spreading post-surgery for measurement^40^. These simulated datasets significantly differ from actual surgery in terms of the background environment, lighting, and other relevant factors present in the surgical videos. Our approach, utilizing gauze images obtained from real surgical environments and constructing an EBL gauze dataset by absorbing actual bleeding into the gauze, led to a model that accurately reflected the real surgical environment and achieved a performance comparable to or better than that of previous studies using human patient datasets.

The limitations of this study are as follows. First, the dataset consisted of gauze images from a porcine experiment and human patients undergoing laparoscopic surgery, which may not fully represent the variability encountered in different surgical scenarios. Further validation with larger and more diverse surgeries is warranted to enhance the generalizability of our model. Second, our current method for estimating blood loss is based on the blood absorbed by the gauze. To predict the overall EBL, future advancements can include the bleeding area segmentation to estimate the volume of intraoperative residual bleeding. In addition, the estimation of the suctioned blood by analyzing the suction bottle images could automate the process of estimating the overall EBL for the entire surgical area. This integration offers a more comprehensive and automated approach to estimate the EBL, enabling more accurate assessments prior to surgery.

In conclusion, the proposed EBL prediction system utilizing P-W CS values accurately measured the intraoperative blood loss, employing crumpled gauze images as the sole input taken during laparoscopic surgery. Validated through a rigorous dual-dataset approach, our automated system provides objectivity and operational efficiency, obviating the need for extraneous activities such as unfolding the gauze or manual weighing. The system’s margin of error, less than 1 g per patient, attests to its reliability and suitability for practical application in real world. With its capability for automated intraoperative blood loss evaluation, the system has the potential to facilitate more informed clinical decision-making. Ultimately, our model aspires to improve both the quality of patient care and the efficiency of perioperative management.

### Supplementary Information

Supplementary Information.

## Data Availability

The data generated and/or analyzed during the current study are available from the corresponding author on reasonable request.
