# Supplementary material for: Automated deep learning model for estimating intraoperative blood loss using gauze images
Source: Sci Rep. 2024 Jan 31;14:2597. doi: 10.1038/s41598-024-52524-3 (PMC10830489; doi:10.1038/s41598-024-52524-3)
Supplement: Supplementary file 1 — Supplementary Information. [file 41598_2024_52524_MOESM1_ESM.docx]

**Supplementary information**

**Automated Deep Learning Model for Estimating Intraoperative Blood Loss Using Gauze Images**

Dan Yoon^1,†^, Mira Yoo^2,†^, Byeong Soo Kim^1^, Young Gyun Kim^1^, Jong Hyeon Lee^1^, Eunju Lee^2,3^, Guan Hong Min^2^, Du-Yeong Hwang^2^, Changhoon Baek^4^, Minwoo Cho^4^, Yun-Suhk Suh^2,5,*^, and Sungwan Kim^6,7,8,*^

^1^Interdisciplinary Program in Bioengineering, Graduate School, Seoul National University, Seoul, 08826, Korea

^2^Department of Surgery, Seoul National University Bundang Hospital, Seongnam, 13620, Korea

^3^Department of Surgery, Chung-Ang University Gwangmyeong Hospital, Gwangmyeong, 14353, Korea

^4^Department of Transdisciplinary Medicine, Seoul National University Hospital, Seoul, 03080, Korea

^5^Department of Surgery, Seoul National University College of Medicine, Seoul, 03080, Korea

^6^Department of Biomedical Engineering, Seoul National University College of Medicine, Seoul, 03080, Korea

^7^Institute of Bioengineering, Seoul National University, Seoul, 08826, Korea

^8^Artificial Intelligence Institute, Seoul National University, Seoul, 08826, Korea

^†^Dan Yoon and Mira Yoo contributed equally as the first authors.

*Yun-Suhk Suh and Sungwan Kim contributed equally as corresponding authors.

**Correspondence to:**

**Sungwan Kim, PhD**

Department of Biomedical Engineering, Seoul National University College of Medicine, 101 Daehak-ro, Jongno-gu, Seoul, 03080, Korea

Tel: +82-2-2072-3126

Fax: +82-2-745-7870

E-mail: sungwan@snu.ac.kr

ORCID: 0000-0002-9318-849X

**Yun-Suhk Suh, MD, PhD**

Department of Surgery, Seoul National University College of Medicine, 101 Daehak-ro, Jongno-gu, Seoul, 03080, Korea

Tel: +82-31-787-7152

Fax: +82-31-787-4078

E-mail: ysksuh@gmail.com

ORCID: 0000-0003-3319-8482


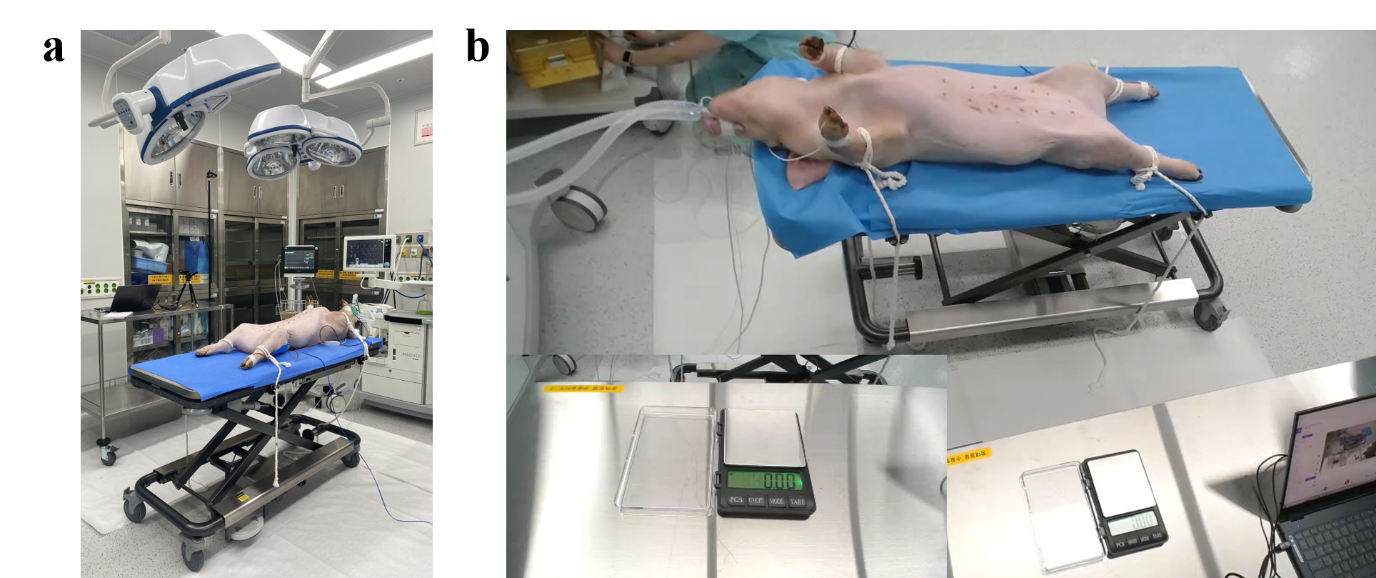


**Supplementary Figure S1**. Description of experimental animal data acquisition. (**a)** Porcine experimental setup for laparoscopic surgery. (**b)** Setup for gauze weight measurement.


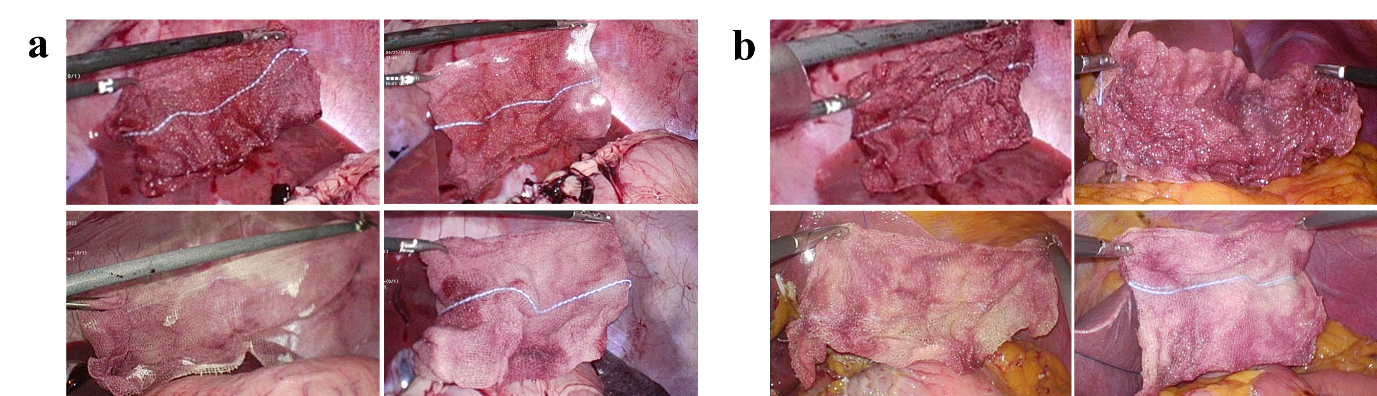


Supplementary Figure S2. Comparison of gauze image data collected for blood absorption: (a) Porcine dataset, (b) Human dataset.

Supplementary Table S1. Performance of the gauze detection model on the test set.

| **Gauze detection performance on the test set (200 gauze patches and 200 background patches)** | | | |
| --- | --- | --- | --- |
| **Model** | **Sensitivity (%)** | **Specificity (%)** | **Precision (%)** |
| **EfficientNet-B3** | 95.0 | 98.5 | 98.4 |
| **EfficientNet-B5** | 96.5 | 98.0 | 98.0 |

**Supplementary Table S2.** Demographics of the patient dataset.

|  | **Patients (N=102)** |
| --- | --- |
| Age (mean ± SD, year) | 62.60 ± 10.48 |
| **Sex** |  |
| Male | 63 (61.8%) |
| Female | 39 (38.2%) |
| BMI (mean ± SD, kg/m^2^) | 24.94 ± 2.98 |
| **ASA score** |  |
| 1 | 25 (24.5%) |
| 2 | 65 (63.7%) |
| 3 | 12 (11.8%) |
| **Charlson comorbidity index** |  |
| 2, 3 | 37 (36.3%) |
| 4, 5 | 54 (52.9%) |
| 6 and more than 6 | 10 (10.8%) |

SD, Standard Deviations; ASA, American Society of Anesthesiologists

**Supplementary Table S3.** Surgical outcomes.

|  | **Patients (N=102)** |
| --- | --- |
| **Type of gastrectomy** |  |
| Distal gastrectomy | 75 (73.5%) |
| Total gastrectomy | 5 (4.9%) |
| Proximal gastrectomy | 5 (4.9%) |
| Pylorus-preserving gastrectomy | 17 (16.7%) |
| **Extent of lymphadenectomy** |  |
| D1 | 3 (2.9%) |
| D1+ | 66 (64.7%) |
| D2 | 27 (26.5%) |
| D2+ | 6 (5.9%) |
| Hospital stay (mean ± SD, day) | 6.27 ± 2.02 |
| Operation time (mean ± SD, minute) | 226.49 ± 50.63 |
| Transfusion during operation | 0 (0.0%) |
| Tumor size (mean ± SD, centimeter) | 2.78 ± 1.94 |
| Number of retrieved lymph nodes (mean ± SD) | 49.66 ± 14.09 |
| **Pathologic stage** |  |
| IA | 77 (75.5%) |
| IB | 7 (6.8%) |
| IIA | 8 (7.8%) |
| IIB | 4 (3.9%) |
| IIIA | 3 (2.9%) |
| IIIB | 3 (2.9%) |

**Supplementary Table S4.** Postoperative complications.

|  | **Patients (N=102)** |
| --- | --- |
| **Early complication** |  |
| No | 89 (87.3%) |
| Yes | 13 (12.7%) |
| **Complication Type** |  |
| Wound | 1 (1.0%) |
| Stump leakage | 1 (1.0%) |
| Motility disorder | 3 (2.9%) |
| Postoperative pancreatic fistula | 1 (1.0%) |
| Intraabdominal abscess | 1 (1.0%) |
| Lung | 3 (2.9%) |
| Urinary | 1 (1.0%) |
| Hepatobiliary | 1 (1.0%) |
| Colitis | 1 (1.0%) |
| **Complication Severity** |  |
| C-D ≥ II | 9 (8.8%) |
| C-D ≥ III | 3 (2.9%) |
| **Late complication** |  |
| No | 102 (100.0%) |
| Yes | 0 (0.0%) |

C-D, Clavien-Dindo
